# Photocatalytic Water Splitting: How Far Away Are We from Being Able to Industrially Produce Solar Hydrogen?

**DOI:** 10.3390/molecules27217176

**Published:** 2022-10-23

**Authors:** Parnapalle Ravi, Jinseo Noh

**Affiliations:** 1 Bionano Research Institute, Gachon University, 1342 Seongnamdaero, Sujeong-gu, Seongnam-si 13120, Gyeonggi-do, Korea; 2 Department of Physics, Gachon University, 1342 Seongnamdaero, Sujeong-gu, Seongnam-si 13120, Gyeonggi-do, Korea

**Keywords:** solar energy, photocatalytic water splitting, hydrogen production, thin films, large scale evolution

## Abstract

Solar water splitting (SWS) has been researched for about five decades, but despite successes there has not been a big breakthrough advancement. While the three fundamental steps, light absorption, charge carrier separation and diffusion, and charge utilization at redox sites are given a great deal of attention either separately or simultaneously, practical considerations that can help to increase efficiency are rarely discussed or put into practice. Nevertheless, it is possible to increase the generation of solar hydrogen by making a few little but important adjustments. In this review, we talk about various methods for photocatalytic water splitting that have been documented in the literature and importance of the thin film approach to move closer to the large-scale photocatalytic hydrogen production. For instance, when comparing the film form of the identical catalyst to the particulate form, it was found that the solar hydrogen production increased by up to two orders of magnitude. The major topic of this review with thin-film forms is, discussion on several methods of increased hydrogen generation under direct solar and one-sun circumstances. The advantages and disadvantages of thin film and particle technologies are extensively discussed. In the current assessment, potential approaches and scalable success factors are also covered. As demonstrated by a film-based approach, the local charge utilization at a zero applied potential is an appealing characteristic for SWS. Furthermore, we compare the PEC-WS and SWS for solar hydrogen generation and discuss how far we are from producing solar hydrogen on an industrial scale. We believe that the currently employed variety of attempts may be condensed to fewer strategies such as film-based evaluation, which will create a path to address the SWS issue and achieve sustainable solar hydrogen generation.

## 1. Introduction

A quick survey was conducted in Scopus to find out how many research projects on water splitting (both with and without sacrificial agents) had been conducted internationally, and a few selected efficient photocatalysts are summarized in Table 1. Despite the rise in reports of solar hydrogen generation over the past two decades, we think the field is still in its infancy and that intense efforts are required to achieve the breakthrough stage. So far, the greatest hydrogen generation efficiency recorded was obtained from a 6 L reactor exposed to UV radiation [1]. The reason why the efficiency of solar light-driven photocatalytic water splitting remained unchanged since the proof-of-concept work published in 1972 is crucial to understand [2]. There are numerous scientific explanations given in the reviews that have recently been published, including an increase in the absorption of light from various solar wavelength regions, manipulation of the band-edge positions of photocatalysts, an extension of the lifetime of photogenerated charge carriers, and the incorporation of photocatalysts with appropriate co-catalysts such as graphene, metals, or metal oxides [3,4,5,6,7,8,9,10,11,12,13,14,15,16,17,18,19,20,21,22]. The current review focuses primarily on several methods for photocatalytic water splitting that have been described in the literature, particularly with a view to increasing hydrogen output and scaling to greater regions when exposed to direct sunlight or one sun.

A viable energy source, hydrogen has the advantages of clean energy, high conversion efficiency, and environmental friendliness. In the previous era, natural gas (or methane) steam reforming has been used to make hydrogen at a low cost, although this process actually produces additional greenhouse gases such as CO_2_ as a byproduct. The 196-nation Paris Agreement, which was signed in April 2016, calls for a decrease in global warming through reduced emissions, which lowers the danger of climate change. As a result, it is critical to switch to using resources that are abundant in nature to generate green and clean fuels. One of the possible routes for the generation of hydrogen in this context is the SWS reaction under direct sunlight. To succeed on a commercial basis, though, there remains a very long way to go. Domen et al. recently outlined one such scenario [30]. It is suggested that 10,000 solar plants, each with a 25 km^2^ surface area and a solar energy conversion efficiency of 10% under AM 1.5G irradiation, would be adequate to provide one-third of the predicted energy needs of the world’s population by 2050. The solar power plants are anticipated to create 570 tons of H_2_ per day from a total projected area of 250,000 km^2^, which is equivalent to 1% of the desert area on Earth. It is essential to mention that the photocatalytic process creating hydrogen mimics natural photosynthesis, in which chlorophyll is known to act as a light absorber. Over the past few decades, numerous photocatalyst materials have been discovered and exploited by researchers in their efforts to generate solar hydrogen efficiently. Throughout the whole literature, the hydrogen evolution rate (HER) of photocatalyst materials in powder form is reported to range from a few hundred mmol h^−1^·g^−1^ to 100 mmol h^−1^·g^−1^. Following are some key findings from a thorough analysis of existing literature reports: (a) When a photocatalyst is suspended, more light scatters than is absorbed, although even low-quality thin films appear to have substantially reduced this issue [31,32,33,34,35,36], thus, making films able to generate additional charge carriers from the first stage of light absorption. (b) Thin films, like solar photovoltaic panels, have a strong potential for scaling up to bigger size panels (supposedly, ~m^2^) and will thus be favored for large-scale hydrogen generation. To create photocatalyst panels, a simplistic painting technique might be used [35,36]. (c) Using certain design features, stirring problems can be resolved with a thin-film/panel technique, which saves energy and is anticipated to reduce material disintegration because the catalyst films will not be subjected to mechanical strain. In this regard, the majority of issues with powder suspension may be solved using films for increased hydrogen production. Based on the various photocatalytic hydrogen generation methods described in the earlier, the present review goes into great detail about the drawbacks of the conventional method of conducting photocatalysis experiments, the benefits of thin film and panel forms of photocatalysts which help to reach commercial hydrogen production, and the underlying science.

## 2. Fundamentals of Water-Splitting Reaction

The water-splitting process entails several photophysical and photochemical stages. Initially, the first process is started by photon absorption, which creates an electron–hole (e^−^-h^+^) pair. The electrons will move to the conduction band and the holes remain at the valance band. The position of the CB minimum (CBM) should be more negative than the water reduction potential of H^+^/H_2_ (0 V vs. RHE), while the VB maximum (VBM) should be more positive than the water oxidation potential of H_2_O/O_2_ (1.23 V). In order to generate more electron–hole pairs, effective light absorption with minimal light scattering is the crucial factor in addition to bandgap engineering of the material [35,37,38]. Moreover, it is the fact that particle catalysts have a considerable light scattering issue rather than an absorption one. The electron–hole pair should be separated and diffuse towards the respective redox co-catalyst sites present in the photocatalyst. Due to (a) charge carriers’ short lifespan and (b) chemical processes’ lengthy time scales, a significant portion of charge carriers (>90%) undergo recombination; this leads to poor net redox reaction, which is one of the major issues of photocatalysis. It should be stressed that the photocatalyst’s function is essential for efficient light harvesting.

General redox reactions that take place to produce oxygen and hydrogen as follows:

(1) $$2H^{+}+2e^{-}\to H_{2}$$

(2) $$2H_{2}O+4h^{+}\to 4H^{+}+O_{2}$$

The main factors increasing SWS efficiency are electron–hole pair separation and diffusion. It should be emphasized once more that charge recombination occurs more quickly than charge usage. To address this issue, the scientific community has created a number of materials and techniques [16].

### 2.1. Hydrogen Generation Using Scavengers/Sacrificial Agents

As previously mentioned, it is essential to separate the electron-hole pairs and diffuse them towards the redox co-catalyst sites to reduce the amount of recombination. In this context, use of sacrificial agents is one of the strategies involved to improve the efficiency. In order to devour the holes, organic molecules are often utilized as the sacrificial agents and involved in photooxidation. In the literature, a plethora of sacrificial agents such as alcohols, amines, and sulphides are used for better HERs [39]. The choice of the sacrificial agent must be carefully considered in order to achieve effective HER. To facilitate the simple electron/hole transfer between light-harvesting photocatalysts and sacrificial reagents, the energy levels (VBM and CBM) of the two materials must match for the sacrificial agent to be chosen. In this context, Na_2_S/Na_2_SO_3_ is used for high HERs in the majority of chalcogenide-based photocatalysts, including CdS, PbS, ZnS, and MoS_2_, which are also known to be visible light-active materials. However, for improved hole utilization and thus superior HERs, TiO_2_ and titania-based composites employ alcohol molecules including methanol, ethanol, and glycerol. However, triethanol amine (TEOA) is used as a sacrificial agent for enhanced HERs in the instance of graphitic carbon nitride (g-C_3_N_4_), which is recognized to be one of the intriguing materials that functions as a visible light-active material with a bandgap of 2.4 eV [8]. However, the quantity of hydrogen produced from the sacrificial agent and water is still up for discussion along with oxidized byproducts such as CO_2_.

Here is the detailed hole utilization mechanism of categorized sacrificial reagents such as alcohols, amines, and sulphides as follows [39,40].

#### 2.1.1. Alcohols (Glycerol):

Glycerol adsorbed on the surface of photocatalyst will interact with holes and forms radicals as shown in Equation (5). Once glycerol engages the holes, the excited electrons on the conduction band consequently produce H_2_ gas as shown Equations (3)–(9). The hydroxyl glycerol in the second stage is unstable, and it interacts with holes, resulting in the formation of glyceraldehyde as shown in Equation (6). Glyceraldehyde dissociates further producing an unstable CO radical that combines with a few holes and eventually converts to CO_2_ as shown in Equations (7) and (8).

(3) $$Metal\;oxide\;NP\;\overset{hv}{\to }h^{+}+e^{-}$$

(4) $$3H_{2}O+3e^{-}\to 3H^{+}+3OH^{*}$$

(5) $$C_{3}H_{8}O_{3}+3h^{+}\to C_{3}H_{5}O_{3}^{*}+3H^{+}$$

(6) $$C_{3}H_{5}O_{3}^{*}+3h^{+}\to C_{3}H_{2}O_{3}^{*}+3H^{+}$$

(7) $$C_{3}H_{2}O_{3}^{*}+2h^{+}\to 3CO^{*}+2H^{+}$$

(8) $$3CO^{*}+3OH^{*}\to 3CO_{2}+3H^{+}$$

Overall net reaction

(9) $$14H^{+}+14e^{-}\to 7H_{2}$$

#### 2.1.2. Triethanolamine (TEOA):

In the case of TEOA as sacrificial reagent, TEOA becomes formaldehyde and then to hydrogen as shown in Equations (14) and (15).

(10) $$Photocatalyst\;\overset{hv}{\to }h^{+}+e^{-}$$

(11) $$2H^{+}+2e^{-}\to \;H_{2}\;$$

(12) $$h^{+}+H_{2}O\to \;\;OH^{*}+H^{+}$$

(13) $$h^{+}+OH^{-}\to \;\;OH^{*}$$

(14) $$TEOA+OH^{*}\to HCHO+NH_{3}$$

(15) $$HCHO+OH^{*}\to H_{2}+by\;products\left(CO_{2}\right)$$

#### 2.1.3. Sodium Sulphide and Sodium Sulphite Mixture (Na_2_S and Na_2_SO_3_):


(16) $$Photocatalyst\;\overset{2hv}{\to }2h^{+}+2e^{-}\;$$

(17) $$At_{CB}\left(2e^{-}\right)+2H_{2}O\to \;H_{2}+2OH^{-}$$

(18) $$At_{VB}\left(2h^{+}\right)+SO_{3}^{2-}+2OH^{-}\to SO_{4}^{2-}+H_{2}O\;$$

(19) $$2SO_{3}^{2-}+2h^{+}\;\to \;S_{2}O_{6}^{2-}$$

(20) $$2S^{2-}+2h^{+}\to S_{2}^{2-}\;$$

(21) $$S_{2}^{2-}+SO_{3}^{2-}\;\to S_{2}O_{3}^{2-}+S^{2-}$$

In this type of systems, sulphide (S^2−^) and sulphite (SO_3_^2−^) can act as sacrificial inorganic reagents for the photocatalytic hydrogen generation because they are very efficient hole acceptors, enabling the effective separation of the charge carriers. The oxidation of S^2−^ and SO_3_^2−^ can occur either by a two-electron transfer process (16) to (18) or one-electron oxidation (19) to (21).

### 2.2. Overall Water Splitting

Overall water splitting (OWS) is an act of producing hydrogen that involves utilizing water as the lone reactant at a pH of 7 with the right photocatalyst under full sunshine. Even if HER and OER happen as shown in Equations (1) and (2), the rate of HER is much lower than it would be with a sacrificial agent. As a matter of fact, the HER values reported from OWS are at least 2–3 orders of magnitude less than those reported using sacrificial agents. The OER (Equation (2)) is a four electron process that cause sluggish kinetics and lowers the total rate of OWS. In this regard, several catalyst materials have been published in the literature with the aim of enhancing the overall kinetics of OWS and so achieving efficient hydrogen production. In this scenario, Domen and colleagues as well as several others have made contributions using a Z-scheme method to effective hydrogen production [17,29,41,42,43,44,45,46]. In which BiVO_4_:Mo and SrTiO_3_:La,Rh used as photocatalyst-I (PS-I) and photocatalyst-II (PS-II), and Au used as electron mediator. The CB of PS-I is closer to the VB of PS-II via Au metallic electron mediator to reduce the resistance to the migration of electrons and holes. Whenever the composite got excited with light energy, both PS-I and PS-II generates photoexcitons and the electrons at CB of PS-I will combined with the holes at VB of PS-II via Au mediator. The terminal electrons and holes will take place in redox reaction. However, compared to the anticipated STH (solar to hydrogen) conversion efficiency values (10% and higher), the efficiency (1%) that has been recorded so far is quite low for commercial-scale hydrogen generation. We think there is a chance to raise the total rate of OWS by employing alternate strategy such as the thin film technique. The efficiency of converting solar energy into hydrogen is often measured using one of two techniques as follows.

(22) $$\mathrm{Apparent}\;\mathrm{quantum}\;\mathrm{yield}\;\left(\mathrm{AQY}\right)=2\times \frac{\mathrm{no}\;\mathrm{of}\;\mathrm{hydrogen}\;\mathrm{molecules}}{\mathrm{no}\;\mathrm{of}\;\mathrm{photons}}\times 100$$

From the above equation, the number of incident photons can be measured using a radiant power energy meter, which considers the following equation (Equation (23)):

(23) $$E=\mathrm{nhv}=\mathrm{nh}\frac{C}{\lambda }$$

where E stands for incoming light energy, n stands for photon number, h for Planck’s constant $\left(6.634\times 10^{-34}\;j\;S\right)$, c for light velocity, and λ for incident light wavelength.

### 2.3. Factors for Achieving the High Efficiency of SWS

It is practically impossible for a single semiconductor material to function as an effective SWS photocatalyst and to carry out all three of the key processes of (a) light absorption from the broad spectrum of sunlight, (b) charge separation and diffusion to redox sites, and (c) actual redox reactions. 

**Figure 1:**
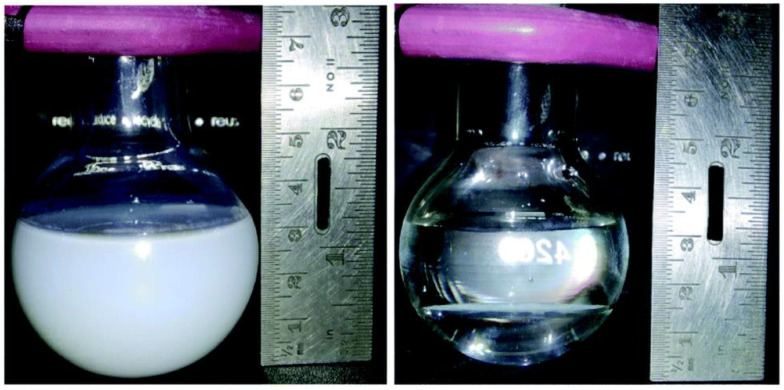
A digital image of a quartz reactor with a capacity of 70 mL (56 mm in diameter at the center) filled with 40 mL of solution and 25 mg of titania-based catalyst powder while it was spinning (left), and in static circumstances, using thin films produced with 1 mg of the same photocatalyst (right). Replicated from ref. [56].

Habitually, a composite photocatalyst made up of at least two (for example, Pt and TiO_2_), three (for example, Au, rGO, and TiO_2_), or more (for Z-scheme photocatalysts) components are effective to move closer to overcome the above-mentioned key processes. Very few researchers have tried to combine the three basic photocatalytic processes using effective synthetic techniques [37,47,48]. Furthermore, a charge diffusion is one approach that might be used to improve the activity by seamlessly combining the charge generating sites and charge usage (or redox) sites [16]. To accomplish this, designing a highly integrated single composite material for effective hydrogen generation from SWS is required. A systematic increase in the complexity of the photocatalyst design of Au-gC_3_N_4_/TiO_2_ (221 mmol h^−1^·g^−1^) outperforms SWS kinetics from g-C_3_N_4_/TiO_2_ (91 mmol h^−1^·g^−1^) [49]. When an integrated Au–N–TiO_2_–graphene composite was used instead of titania alone, the HER of 525 mmol h^−1^·g^−1^ was found [50]. The improvement in activity with 1275 mmol h^−1^·g^−1^ and sustained activity for 125 h were attributed to the electronic integration of an Au–Pt bimetal cluster with titania [51]. On titania, a similar observation was achieved using Au–Ag or Au–nanorod [52,53]. According to a recent study, a single titania nanotube material with heterojunctions between native and nonnative structures, such as the presence of the anatase, rutile, and brookite phases, and Pt as the co-catalyst exhibited higher HER activity from SWS under one sun conditions than anatase-rutile (2.5 mmol h^−1^·g^−1^) or bare anatase (0.15 mmol h^−1^·g^−1^) [54,55]. The difference in activity is attributable to the existence of bulk heterojunctions in a composite material as opposed to a single junction (anatase-rutile) or bare anatase nanotubes, which can aid in effective charge transfer between the particles. Independent of the photocatalyst systems, the outcomes given here suggest that SWS activity increased from a negligible value to 7.6 mmol h^−1^·g^−1^ [47,48,49,50,51,52,53,54]. Furthermore, the HER was measured using a photocatalyst made of Au–Pd/rGO/TiO_2_ both in particulate and thin-film forms by easy casting on a glass plate. The measured HERs from thin-film and particulate versions are 21.5 and 0.50 mmol h^−1^·g^−1^, respectively. It is specifically due to the difference of light absorption. Despite having all of the necessary photocatalyst components, the HER values are incredibly low for particulate photocatalyst systems and the evaluation of HER in the particulate form of the photocatalyst composite acts as a unifying factor among all these findings. Simple comparisons between photocatalyst suspension and the identical photocatalyst preserved in an aqueous methanol solution as a thin film demonstrate (Figure 1) that the former exhibits low light penetration while the later exhibits high light penetration [56]. Regardless of the angle of light incidence, light penetration would be inadequate with photocatalyst suspension. 

Although there are multiple reasons that prevent SWS from operating at a high efficiency, major SWS limiting issues and some potential solutions are provided as listed below:

(a) The primary limiting element is the very different time scales between photophysical and photochemical processes and how to connect them. Structural and electronic components integration is crucial to be optimized for efficient diffusion of charge carriers to redox sites and their exploitation for redox reactions. Quantum dots (QDs) and 2D-layered materials might be used to overcome the aforementioned issue. It is also important to note that the nanoscience, which only involves photophysical processes, has made a significant contribution to the rapid expansion of applications involving light emission. It is very desirable to use synthesis methods that would result in bulk heterojunctions in a photocatalyst composite. For instance, the assembly of QDs in the pores of wide bandgap materials using techniques such as SILAR (successive ionic layer adsorption and reaction) results in bulk heterojunctions.
(b) In general, scaling up the catalyst quantity, even from 10 to 100 to 1000 mg at a laboratory level, substantially reduces the effectiveness of any photocatalyst system. This is largely because of poor light absorption combined with excessive charge recombination [57]. The current review tackles this issue in depth, through the use of a photocatalyst thin film or a panel with increased hydrogen generation.
(c) Since OWS is often carried out in extremely acidic environments [58], it is essential to be able to conduct the tests at a pH close to neutral (pH = 7). There is definitely a need for greater study in this area.
(d) To the best of our knowledge, noble metals are frequently used, which is not a cost-effective solution, and there is no reasonable consideration given to the selection of a certain co-catalyst for a specific semiconductor [58]. More research must be done to examine more affordable and plentiful co-catalysts, with a focus on rational selection.
(e) It is necessary to switch to using environmentally beneficial and/or biomass-derived materials such as glycerol and cellulose instead of sacrificial ones such as methanol. Sacrificial agent use in water splitting may be a temporary fix, and OWS will be the long-term fix [39].
(f) Despite the fact that CdS, PbS, and other chalcogenide QDs have very strong visible light absorption qualities and the capacity to control the bandgap, they are also vulnerable to photo-corrosion and are unfriendly to the environment [59,60]. The oxide-based QDs should be the focus of additional efforts.

Until now, a lot of work has gone into creating effective systems for the SWS process, which produces hydrogen. However, none of the photocatalysts can produce hydrogen in a practical manner. Indeed, under sunshine or one-sun circumstances, none of the powder catalyst systems have been studied at the gram scale. Table 2 lists some of the top hydrogen production activity values obtained using several powder-based catalysts. 

The current analysis concentrates on the additional crucial factors that contribute to improving the overall effectiveness of solar light-driven water splitting. The current study stresses a thin film-based technique. It is advantageous to boost efficiency as opposed to the particulate-based research that is commonly used by many researchers worldwide. 

#### 2.3.1. Mechanical Stirring Is Unfavorable

It is difficult to analyze the photocatalyst powder on a big scale, and continual stirring of the powder solution is necessary to enhance light harvesting. It is an energy expensive when mechanical churning is used at such huge loads. Following the conclusion of reaction investigations utilizing photocatalyst powders, the collection of the catalyst using centrifugation and filtering is another time-consuming and energy-intensive step [71]. Above all, the essential notion of better light absorption does not appear to happen with an increase in the photocatalyst quantity in the suspension. Additionally, when the catalyst concentration rises, light cannot reach all areas of the solution due to the turbidity of the solution. Moreover, the price of hydrogen generated using traditional steam reforming techniques is $3–4 per kg, therefore any new approach needs to at least be competitive with that to be taken into consideration. The typical thin film method of producing solar cells would be advantageous for better light absorption, and many benefits and drawbacks of photocatalysts in their thin-film and particulate forms, respectively, will be examined in this context.

#### 2.3.2. Loading Effect

It is anticipated that more catalyst will need to be loaded into the solution in order to produce solar hydrogen on a wide scale. In this context, Maeda et al. examined the impact of loading on the quantum yield of hydrogen for a Z-scheme photocatalyst using Pt/ZrO_2_/TaON as the hydrogen evolution catalyst and Pt/WO_3_ as the oxygen evolution catalyst [57]. It was discovered that the AQY drops from 6.3 to 2.7% when the total catalyst dosage rises from 75 to 150 mg. In contrast to the predicted rise in light absorption with an increase in the quantity of powder catalyst loading, only a portion of the particles are exposed to photons at a given moment, and the remainder particles are inactive. However, when there is only a tiny quantity of catalyst present in the test solution, the majority of the particles are exposed to photons and form a greater number of charge carriers, which helps to achieve high efficiency. This is directly corroborated by the research done by Nalajala et al., who found that using 25 mg of powder Pd/TiO_2_ in 40 mL of aqueous methanol produced less H_2_ (9 mmol h^−1^·g^−1^) than using 1 mg of the same catalyst under the same circumstances (32 mmol h^−1^·g^−1^) [34]. The hydrogen yield (HY) per gram of catalyst in water splitting studies with a tiny quantity of catalyst (1 mg) would appear to be quite high (Table 3). Reporting the hydrogen evolution rate for a few different weights for a certain volume of water or aqueous solution is the preferred method, since it enables other labs to replicate the findings. However, this raises the unavoidable question of what is the best method to use for handling massive quantities of catalyst for SWS.

#### 2.3.3. Scale-Up and Disintegration Issues of Photocatalysts

Under reaction circumstances, the catalyst system must remain intact in order to produce hydrogen from SWS sustainably. The catalyst system in particular is anticipated to be stable for prolonged exposure to light and a wet environment. The development of photocatalyst materials in the particulate form is now the focus of several initiatives. The greatest recorded hydrogen production activity using various catalyst systems are shown in Table 2; However, they were selected because (a) they could demonstrate sustainability for at least 10 h in one day of sunlight and (b) the catalyst quantity used in real studies needed to be at least 10 mg. Whatever the provided catalysts were confirmed to be active, only a small number of investigations have demonstrated their stability for 100 h or more [65,70]. A contrasting truth may be found by simply comparing the findings provided in Table 2 and Table 3. Higher HER values were seen with 1 mg of catalyst from the actual data (Table 3) compared to those shown in Table 2, which use 10–200 mg of catalyst. As shown in Figure 1, a suspension form of a catalyst cannot increase activity linearly with an increase in catalyst amount. A few studies also indicate a decline in activity within 10 h after the reaction, raising the likelihood of catalyst breakdown [61]. The activity and stability of the thin film were established by Schroder et al. [35], during the 30 days active light period, and Goto et al. investigated the films for 1000 h [36]. A stable photocatalyst would sustain HER activity for a very long time.

## 3. Light Absorption and Scattering

While there are several semiconductor materials with adequate band gaps and band edge locations for SWS, effective light absorption in their powder state is a significant problem. According to the Beer–Lambert equation, the absorption coefficient that depends on wavelength and material thickness determines how much light is attenuated. However, it is typically believed that enough light absorption in the normal UV-Vis absorption spectrum is required for its effective light absorption properties. In actuality, smooth, high-quality solid surfaces absorb solar light more effectively than rough, poorly absorbent particle surfaces.

**Figure 2:**
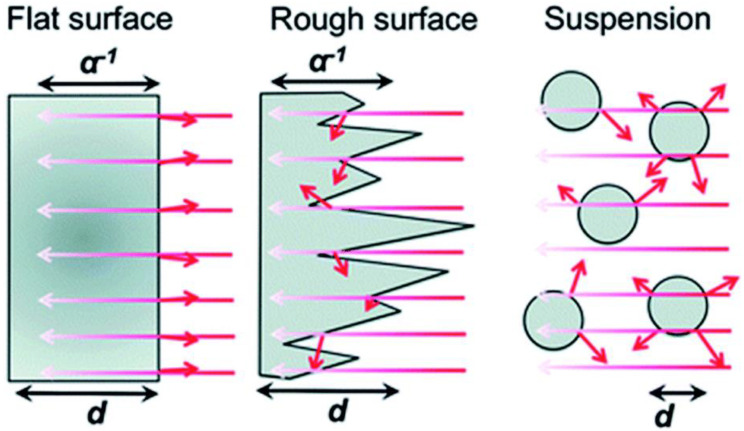
Dispersion of light in nanostructured and flat films as well as in suspensions of particles. A α^−1^ is the optical penetration depth, and d is the film or particle thickness. Short arrows denote light that has been reflected or dispersed. Replicated from ref. [13].

As seen in Figure 2, light is primarily dispersed from all sides of particles (in suspension) as opposed to thin films, which typically absorb light due to their comparatively flat surfaces and high absorption coefficients. Thin films can actually absorb more light with a 50–500 nm uneven surface structure than with a pristine smooth surface, which is undesirable since it would reflect more light, and an anti-reflective coating used in solar cells would be necessary for very flat surfaces. It is anticipated that the thin film-based photocatalyst would yield more charge carriers than the particulate catalyst [6]. When compared to the intensity at the material’s surface, light penetration depth (also known as skin depth) is described as a drop in light intensity to 37% (or 13%). The same reasoning holds true here as they did in the case of dye-sensitized solar cells (DSSCs), where this feature has been well investigated. For maximal incoming light absorption, a thickness range of 8–12 μm is recommended. The decreased thickness of the material in a film with a thickness of less than 8 μm results in less light absorption. Whereas, due to the additional layers of materials present at the bottom FTO/ITO plate for solar cell applications, films thicker than 12 μm result in increased recombination as the charge carriers must diffuse across these layers.

## 4. Towards Enhanced Hydrogen Production with a Thin-Film Approach

The advantages, production methods for large-scale thin films, their photocatalytic activities, stability factors, and the re-absorption of emitted light for better activity are all covered in this part along with other procedures that are successful for SWS.

### 4.1. Thin Film Approach

By placing active photocatalysts as films over a fixed substrate, a considerable number of problems with the particulate form of the catalyst may be resolved. Better light absorption may be anticipated than with equivalents in the solution that are suspended powders since the catalyst is attached to the substrate with the specified thickness. Use of centrifugation for gathering the suspended powders or mechanical stirring for better dispersion will be avoided. Small volumes of liquid may be used to operate thin films for reactions, which is unquestionably a procedure that uses less time and energy. Furthermore, this method may be an economically feasible choice for sustainable hydrogen generation due to the minimal infrastructure (little number of materials and no expensive equipment needed) and simplicity of scaling up.

#### Advantages of Thin Films over Particles for Photocatalysis of Hydrogen Production

It is vital to draw attention to a number of benefits that a thin film form has over a particle form. The following details are noteworthy:

(i) Light absorption: As illustrated in Figure 1 and Figure 2, films rather than particle suspensions are more commonly used in the initial stage of effective light absorption in photocatalysis. Thin films produce a significant number of charge carriers by efficient and consistent light absorption. Thin films allow for the generation of many charge carriers since the photocatalyst is fully and evenly exposed to the photon source during the whole time period. This is clear from the video depiction of solar hydrogen that is discussed in the literature [34].
(ii) Maximal activity, little input, and continuous process: It should be stressed that there is a significant decrease in hydrogen production in photocatalysis studies with a particulate suspension as catalyst concentration rises. High hydrogen yields are produced using a thin film that is the ideal thickness (8–12 μm). The maximal activity with the least amount of material is shown by up to an order of magnitude and greater activity recorded with the same quantity of the substance in thin film form as opposed to powder. Additionally, as opposed to a batch procedure using suspension, the thin film makes the process continuous.
(iii) Energy saving: Due to the lack of mechanical stirring, which may be replaced by centrifugation for catalyst removal in the following batch or recycling, it is anticipated that thin film-based solar panels would have much lower operating costs. However, they will involve suspension, thus there is a cost/energy consideration.
(iv) Running cost: It should be noted that solar hydrogen production with thin films is also possible with water (or solution) layers as thin as 1 mm, and hydrogen bubbles are discharged smoothly. However, it is possible to further reduce the water layer thickness to a few nanometers. This will be crucial for using extremely effective catalysts, which would continuously and without resistance create hydrogen bubbles. The smooth and immediate release of bubbles is made possible by the thinness of the water layer. It should be noted that delayed bubble development reduces the catalyst’s ability to absorb light since bubbles have a strong tendency to disperse light.
(v) Coalescing diverse material components: The artificial leaf and QuAL ideas propose to combine many elements, such as co-catalysts for reduction and oxidation processes and various light-absorbing quantum dots in one device. Therefore, this method takes care of the (structural and maybe electronic) integration of various material components, whereas it is challenging to evenly include all of the aforementioned elements throughout the particle bulk catalyst.
(vi) Mass transfer issues: The greater engineering problem is distributing the reactant(s) equally across the film/panel device. However, since there is no need for pressure and hydrogen may be produced with or without scavengers in merely a millimeter of liquid water thickness, a simplistic tilting mechanism may be used to circulate the solution using gravity. In addition, tilting and sun tracking might be properly integrated to increase hydrogen generation.

### 4.2. Scaling Up and Thin Film Preparation Techniques

Thin film technology is well recognized to have many uses in diverse science and technology fields, including solar cells, optics, electronics, and many more. Low material usage and the frequent use of flexible substrates are benefits of thin-film devices that are difficult to obtain when applying bulk materials directly. It should be noted that thin film characteristics and activity are frequently influenced by the type of deposition technique. Because of uncomplicated, inexpensive, and convenient features for depositing a wide range of materials, chemical techniques are often effective for the deposition of large-area thin films. Although there are several photocatalysts available to produce the solar hydrogen, the discoveries made in the lab must be scaled up. In this context, a few techniques, including spray coating, slot-die coating, and screen printing, are mentioned in the literature to create films of various diameters [82]. For the film preparation using particle precursor photocatalysts, there are just a few reports accessible at this time. The following section contains some literature reports on various photocatalysts prepared as thin films using drop-cast and particle transfer techniques.

#### 4.2.1. Drop-Cast Method

A thin layer of a Rh_2−y_Cr_y_O_3_/(Ga_1−x_ − Zn_x_)(N_1−x_O_x_) photocatalyst was created by Xiong et al. using a drop casting approach on a flat piece of frosted glass [83]. The researchers added silica powder with different particle sizes (from nanometers to micrometers) to the photocatalyst in order to control the porosity and hydrophilic character of thin films. To create a homogeneous suspension, the photocatalyst powder (20 mg) was sonicated in 200 mL of deionized water. A quantity of 20 mg of silica powder was added with the aforementioned combination and held for sonication in order to better understand how the photocatalyst’s porosity and hydrophilic nature affected its performance. The suspension was then dropped onto a clean, frosted glass plate and held there to dry while it was heated to 323 K. In order to create a film with a consistent thickness, this operation was done ten times.

**Figure 3:**
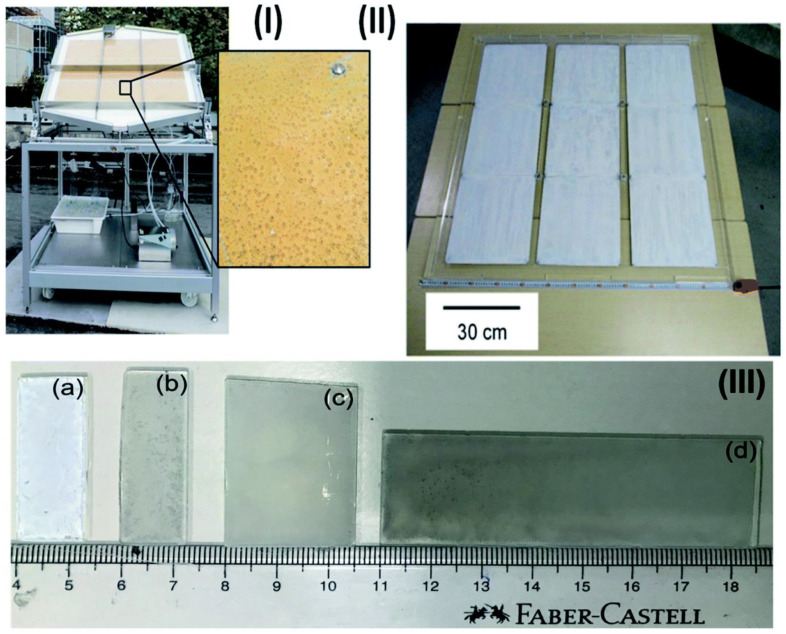
Images of panels and thin sheets in various sizes: (**I**) picture of the large-scale hydrogen generation system for photocatalysis illuminated by the sunshine. Nine stainless steel plates, each measuring 28 × 30 × 0.1 cm^3^, make up a photocatalyst (Pt@mp–CN) panel. The mesoporous carbon nitride photocatalyst emitting hydrogen bubbles can be seen when magnified. (**II**) Nine 33 × 33 cm^2^ sheets make up a 1 × 1 m^2^ SrTiO_3_:Al photocatalyst panel. (**III**) Sizes of thin films (**a**) 1.25 × 3.75 of P25 (**b**) 1.25 × 3.85, (**c**) 2.5 × 3.75, and (**d**) 2.5 × 7.5 cm^2^ of Pd/P25. Replicated from ref. [36].

In a different study by Schroder et al., a Pt@mp–CN (Pt cocatalyst on mesoporous carbon nitride) photocatalyst was immobilized on a stainless-steel plate by a drop casting methodology over different substrate dimensions, such as 3.5 × 3.5 × 0.25 cm^3^ for the laboratory reactor and 30 × 28 × 0.1 cm^3^ for the demonstration reactor, as shown in Figure 3I [35]. Under direct sunlight, the photocatalyst panels were employed to produce solar hydrogen. It is important to note that the negative zeta potentials of both Pt and carbon nitride caused Pt nanoparticles to be manufactured separately by a microemulsion process [84].

By using a similar approach to Schroder et al., Goto et al. developed a 1 × 1 m^2^ size SrTiO_3_-Al panel (Figure 3II) for large-scale hydrogen production [36]. The study focused on a number of factors to enhance the performance of panel-type catalysts for water splitting reactions, including tilting angle (10–20°) and water layer thickness (1–5 and 5 mm). These factors are crucial to reduce liquid weight and, consequently, pressure, in order to achieve the smooth release of gas bubbles. Without induced convection, a layer of 1 mm thick water might release hydrogen bubbles.

Prominently, this study showed that crack development did not impair hydrogen production in contrast to PEC cells and PV-based electrolyzers, where photocatalyst sheets do not form a series circuit in the panels [36]. The constructed photocatalyst panels were examined for H_2_ production from pure water under both artificial and natural light sources.

In a recent study, Nalajala et al. developed thin films of titania-based photocatalysts (Pd/P25) on glass plates and assessed their hydrogen generation capabilities in the presence of direct sunlight. The creation of diverse sized and shaped thin films was accomplished using a drop-cast process (Figure 3III). They employed methanol as a sacrificial agent, Pd as a co-catalyst, and P25 (TiO_2_) as a light-harvesting material to create hydrogen in their experiment. Similar to the previous research by Goto et al., the thin films showed micron-sized fracture development, although this did not impair the thin film’s functionality.

#### 4.2.2. Particle Transfer Method

**Figure 4:**
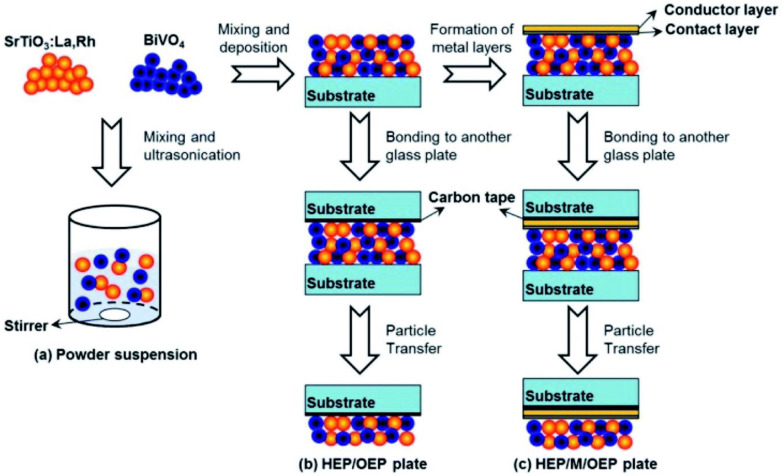
An illustration showing how photocatalyst particles are transferred across glass substrates to create photocatalyst sheets: (**a**) preparation for powder suspension, (**b**) preparation of HEP/OEP plates on carbon tape and without a metal layer, and (**c**) preparation of HEP/M/OEP plates with sandwiched metal layers on carbon tape. Replicated from ref. [41].

For the creation of thin films from powder catalyst suspensions, the particle transfer (PT) method was developed. In this procedure, a combination of photocatalyst granules was dispersed in isopropanol and then applied to clean glass plates measuring 3 × 3 cm^2^. A thin layer of catalyst film was created over a glass plate by quickly drying the catalyst suspension after it had been deposited (Figure 4). The contact layer that will make contact with semiconductor particles was created using a vacuum evaporation process. As a contact layer, any of the metals (Au, Ag, Al, Ni, or Rh) can be employed. Then, using a vacuum evaporation process, an additional Au layer was deposited over the previously developed contact layer of the film in order to provide the prepared film with the necessary conductivity and mechanical stability. Taking off the first or primary glass plate, the metal film that makes up the particle photocatalysts was adhered to a second glass plate using carbon tape. To remove any extra particles that had accumulated on the photocatalyst layer, the resultant glass plate underwent a brief ultrasonic treatment. 

Domen and coworkers used this PT approach extensively and published their findings for a range of particle photocatalyst materials [29,41,42,43,44,45,84,85,86,87]. The possibility of destroying thin films when removing (lifting off) the main glass plate is a major worry with this method of preparation. In order to facilitate the conductivity between the HEP and OEP particles, it is also important to take into account that a complicated equipment needed to generate the metal contact layer.

#### 4.2.3. Screen Printing

**Figure 5:**
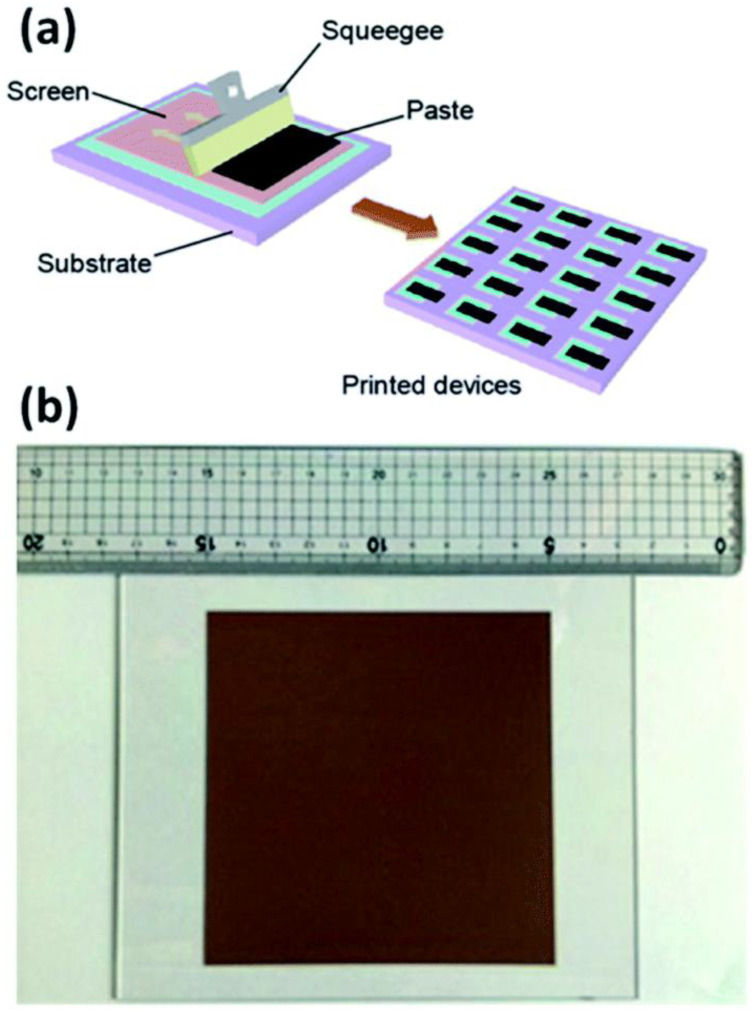
(**a**) Schematic for screen printing method, and (**b**) photo image of a 10 × 10 cm^2^ screen printed device. Replicated from ref. [82,29].

One of the reliable methods that offers large-scale thin films with maximal material usage is screen printing. The drying and/or sintering processes were crucial to dictate the production capacity of screen printing rather than the screen-printing method itself. Hu et al. showed that the screen printing (Figure 5a) can give the cells of 10 × 10 cm^2^ size with an efficiency of 10% and exhibited its stability under light for 1000 h, stability outside for 30 days, and stability over a year of storage [88].

This method was also developed by Wang et al., who used a powder photocatalyst to generate large-scale thin films [29]. In their investigation, printing ink was made from a combination of organic binders, catalyst powder (SrTiO_3_:La, RhBiVO_4_:Mo), and Au colloid solution in ethanol. The paste was screen printed on a 3 × 3 cm^2^ glass substrate after the ethanol had evaporated. Furthermore, this method may also be used to create a 10 × 10 cm^2^ photocatalyst sheet (Figure 5b) with various Au concentrations (up to 40 wt.%). As a result, this method also showed that systems for large-scale hydrogen synthesis in the presence of direct sunlight are possible. It should be noted that large-area solar cells, particularly for DSSCs, were produced using the screen-printing technique [89].

#### 4.2.4. Doctor Blade Method

The doctor blade approach is a well-known and popular technique for creating a homogeneous covering of broad bandgap semiconductor layers for dye-sensitized solar cells (DSSCs). A well-mixed slurry of target materials and additives suspended in a solvent was applied on an appropriate substrate (often an FTO or ITO plate). A doctor blade was then continuously moved across the substrate. Then the slurry spreads across the substrate and a high-quality thin layer is created after drying (at 400 °C for TiO_2_ films). Different micron-thicknesses of thin films can be produced according to the quantity of materials used, the size of the substrate, and the speed of the doctor blade. High quality thin films are likely to be produced when the right additives and solvents are used in a slurry under the best possible circumstances. For modest size, up to 10 cm^2^, this method of preparation is effective at a laboratory level. However, as already noted, it may be expanded to a bigger extent, perhaps in conjunction with other techniques, so it is worthwhile to further investigate. N-doped mesoporous titania sheets (TiO_2−X_N_X_) production for DSSC applications was described by Sivaranjani et al. [6]. Patra et al. [60,90] described a quasi-artificial leaf device that was made using AuTiO_2_ thin films employing the doctor blade approach and was then sensitized with chalcogenides (CdS, PbS, and ZnS) using the SILAR method. We believe the SILAR approach should be expanded to build active catalytic components on porous supports, which is not feasible with any current techniques.

### 4.3. Effect of Binders in Thin Films

For making thin films, organic and inorganic binders are frequently employed in order to maintain the thin films in place on the surface of the substrate and avoid peeling. However, employing binders might sometimes result in undesirable characteristics or actions. The following is a list of potential outcomes: (a) Due to high temperature calcination, organic binders employed in the doctor blade process, such as cellulose, are often eliminated [40,60,90]. The porosity of the thin films is preserved when binders are removed at high temperatures. However, if an organic binder is left in place, the presence of reactive oxygen species in application circumstances may cause them to oxidize [91]. In order to create stable and uniform thin films, Nafion was utilized as a binder [35]; in fact, Nafion serves to prevent the separation of meso porous carbon nitride (mp-CN) from the stainless steel substrates. Furthermore, it should be noted that Nafion possesses effective proton conductivity, which actually reduces the mass transfer resistance inside the catalytically active layer. (b) SrTiO_3_–Al thin films were created on glass substrates by using inorganic binders, 20 nm SiO_2_ nanoparticles [36]. Silica nanoparticles’ primary function was to create thin coatings with excellent adhesion to the substrate. The catalyst and silica (1:2) solution was coated, dried, and then calcined at 623 K to produce films that were 10–20 microns thick. (c) It must be emphasized that thin films with good stability may still be created without binders and can be tested for hydrogen production. In fact, we advise to use this technique to assess the performance of any photocatalyst in thin film forms. The stability of the film should be improved by the inclusion of binders, which is crucial for HER research. Binders should be carefully selected such that their role does not hinder the underlying activity but rather enhances it. If the binder is included in the final photocatalyst thin film, it must be chemically inert and incapable of reacting with charge carriers or with water in an aqueous media when light is present. The amount of binder applied must also be tuned for every photocatalyst system because one binder does not necessarily work for all semiconductors or composites.

### 4.4. Photocatalytic Activity of Sheets and Films

Recently, Xiong et al. demonstrated the photocatalyst panels of Rh_2−y_Cr_y_O_3_/(Ga_1−x_Zn_x_)(N_1−x_O_x_) consisting of (Ga_1−x_Zn_x_)(N_1−x_O_x_) main catalyst and Rh_2−y_Cr_y_O_3_ cocatalyst [86]. In this investigation, using several methods including squeegee and drop-casting processes, the catalyst and silica (micron size) combination was applied to 25 cm^2^ frosted glass plates. The OWS activity of the panel-type catalyst yielded a determined HER (9 mmol h^−1^·mg^−1^) that is equivalent to HER rate (11 mmol h^−1^·mg^−1^) from the standard technique of assessing photocatalysts in powder form. It was also discovered that the problem of transport of water and product gases via the interparticle void spaces is avoided by the inclusion of micrometer-sized hydrophilic SiO_2_ particles. It should be noted that photocatalyst particles range in size from 200 to 400 nm, and that the absence of silica in the photocatalyst layers makes water diffusion challenging. From the above, the ability to scale up thin films and a reduction in mass transfer issues are the key benefits.

Wang et al. showed high activity using semiconductors immobilized as thin films on metal layers for Z-scheme water splitting (Figure 4); particulate BiVO_4_ (OEP) and SrTiO_3_:La,Rh (HEP) semiconductors [41]. In the absence of redox mediators, the activity of the photocatalyst in thin film form with an Au layer between them is discovered to be 4.5 mmol h^−1^·cm^2^, which is 6 and 20 times greater than that of their comparable powder counterparts (0.8 mmol h^−1^·cm^2^) and without metal layers (0.2 mmol h^−1^·cm^2^), respectively. The constructed system’s remarkable performance is related to the ease with which electrons may move between BiVO_4_ and SrTiO_3_:La,Rh through the metal layer (Au). Since both HEP and OEP are presented close to one another, a drop in H^+^ and OH^−^ overpotentials has also been noted. As a result, the study focused on how one might increase activity using film-based systems by facilitating efficient charge transfer via metal layer between the photocatalysts of the HEP and OEP, which in reality give correct contact as well as retain the particles intact with one another. For better activity of photocatalyst sheets linked to metal layers, Wang et al. conducted a research on Mo- and La-doped BiVO_4_ (BiVO_4_:Mo) particles encased in a gold (Au) layer [29]. The above Z-scheme catalyst complexes demonstrate 1.1% STH conversion efficiency with an AQY of 30% using monochromatic light of 419 nm and pure water of pH = 6.9. Furthermore, it has been demonstrated that the surface modification using Cr_2_O_3_ and annealing the system at 573 K for 20 min may be used to optimize the electron transport of SrTiO_3_:La, Rh/Au/BiVO_4_:Mo and reduce side reactions, respectively. Since the production of H_2_ and O_2_ occurs near together, a significant backward reaction results, which needs to be reduced for the developed photocatalyst sheet systems to function more effectively. By employing carbon as a conducting layer instead of Au, these issues may be solved. Another study used rGO as a conductive binder to increase the photocatalyst sheets’ ability to split water [84].

Due to the ability to absorb visible light and improve solar hydrogen evolution, oxysulfides are considered to be a viable HEP component of the Z-scheme photocatalyst. In this scenario, Sun et al. recently showed a photocatalyst sheet using the compounds of La_5_Ti_2_CuS_5_O_7_ (LTC) and BiVO_4_ as the HEP and OEP, respectively [43]. In this work, p-type doping (Ga^3+^, Al^3+^, Sc^3+^, and Mg^2+^) at the Ti sites and the production of La_5_Ti_2_Cu_0.9_Ag_0.1_S_5_O_7_ (LTCA) solid solution for the OWS reaction greatly increased the activity of this system. When LTC/Au/BiVO_4_ was in powder form in pure water, no activity was seen. However, when LTC/Au/BiVO_4_ was in sheet form and exposed to visible light, substantial activity (0.47 mmol h^−1^·mg^−1^) was seen. In pure water under visible light, Ga-LTCA/Au/BiVO_4_ had more activity (2.2 mmol h^−1^·mg^−1^) than LTC/Au/BiVO_4_. Ga^3+^ is demonstrated to be a superior dopant to increase the activity of the sheet among the p-type doping elements.

**Figure 6:**
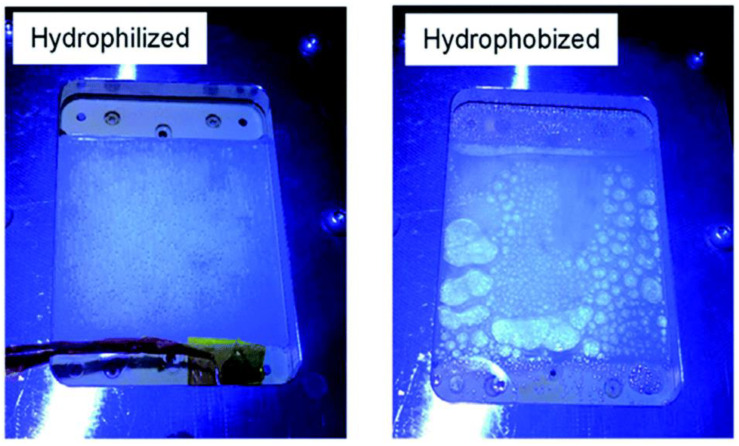
Photos taken while being illuminated via hydrophilic and hydrophobic windows. Replicated from ref. [36].

A panel-type reactor was created by Goto et al., in which the Al-doped SrTiO_3_ catalyst was applied as a thin layer on a glass plate using a drop casting process and tested for OWS reaction. Doping Al^3+^ into the starting material is crucial for improving SrTiO_3_ activity and controlling particle development. Two significant parameters that were examined during the investigation are hydrophobicity and hydrophilicity of the inner surface of the window that was covered over the reactor setup (Figure 6). It has been discovered that the hydrophobic properties of the window allow gas bubbles to develop and proliferate (Figure 6), although they have no effect on the panel’s overall functionality. In order to prevent the buildup of potentially explosive H_2_ and O_2_ gas combination bubbles inside the reactor, it is advised that the hydrophilic nature of the window be used. Under natural sunlight on clean water, the 1 × 1 m^2^ flat photocatalyst panel displayed the STH of 0.4 percent. High rates of H_2_ and O_2_ were found at 5.6 mL. h^−1^·cm^−2^, which corresponds to a smooth release of bubbles from the panel and a STH of 10%. Here, it should be noted that elevating light output was required to reach the STH of 10%. The overall view of comparison between thin-film systems and powder form systems are given in the Table 4.

### 4.5. Quasi Artificial Leaf Concept with a Novel Design of Solar Cell Materials and Devices

**Figure 7:**
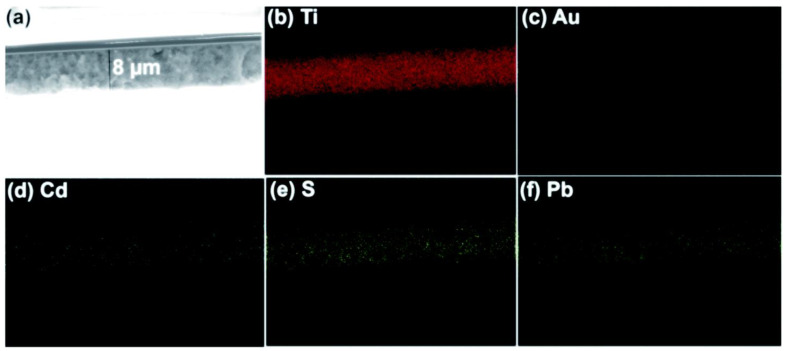
Bulk heterojunctions are demonstrated throughout the thin film employing (**a**) FESEM, and EDX chemical mapping analysis of (**b**) Ti, (**c**) Au, (**d**) Cd, (**e**) S, and (**f**) Pb. EDX result shows the uniform distribution of chalcogenides. Replicated from ref. [60].

The idea of an artificial leaf is widely recognized for dividing water. Trevisan et al. and Patra et al. used the quasi-artificial leaf (QuAL) concept to produce hydrogen [59,60,93]. Here, certain charge carriers such as electrons are collected and used at the Pt-co-catalyst sites, and holes are directly introduced into the solution to oxidize sacrificial agents. Based on the QuAL theory, porous titania (also known as Au–TiO_2_) and Pt were used to create a solar cell, and a SILAR approach was used to build PbS and CdS quantum dots in situ within the pores of titania. The FESEM-EDX chemical mapping was used to show the distribution of PdS and CdS quantum dots in the solar cell from the top to bottom layers of titania (Figure 7). The key benefit of employing the SILAR technique (assembly QDs) in this system is the production of many bulk heterojunctions between chalcogenide quantum dots and a porous Au–TiO_2_ matrix. Despite having a total composition of only 3%, chalcogenide is evenly distributed throughout the photoanode’s porous matrix, which greatly aids in the efficient conversion of light into chemical energy.

The following is two key benefits of the QuAL solar cell: (a) The whole region of UV and visible light as well as a little portion of the NIR range is completely absorbed by a combination of distinct band gap semiconductors and nano gold. (b) The QuAL device operates at zero applied potential and doesn’t require any potential. In this instance, 1 cm^2^ was covered with 2 mg of photoanode material (AuTiO_2_/PbS/CdS), which produced 12 mL.h^−1^ H_2_ under one sun circumstances. The results might be linearly extrapolated to large photoanodes, such as 23 × 23 cm^2^ and 46 × 46 cm^2^ result in 6 and 24 L.h^−1^ H_2_, respectively.

**Figure 8:**
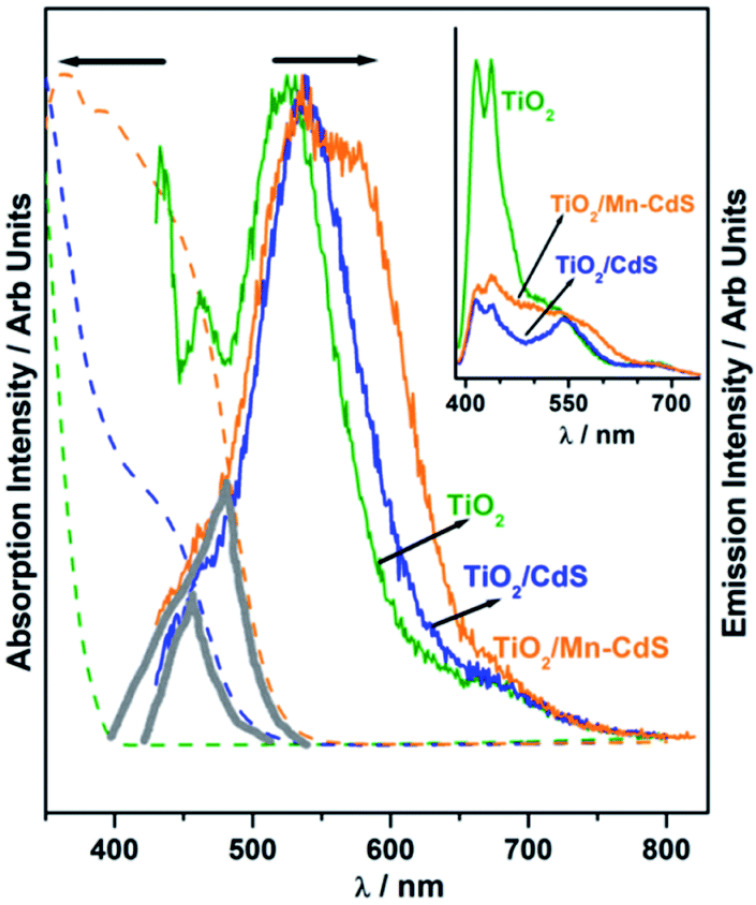
Photoluminescence spectra of TiO_2_, TiO_2_/CdS, and TiO_2_/Mn–CdS obtained with a 420 nm excitation source; emission characteristics are normalized. Additionally, photoluminescence is compared to the UV-visible absorption spectra that were obtained for all three materials (shown as a dashed line in the same color). In the picture, a solid triangle area emphasizing the secondary light source accessible for chalcogenide absorption highlights the wavelength range where the absorption and emission spectra coincide. The photoluminescence spectra of TiO_2_, TiO_2_/CdS, and TiO_2_/Mn-CdS, which were obtained at a wavelength of 370 nm, are displayed in the inset. Replicated from ref. [93].

Patra et al. employed a cutting-edge concept to significantly expand the QuAL concept’s ability to generate more hydrogen. Due to the extensive charge recombination, the photocatalyst’s efficiency is inevitably low and frequently results in radiative emission, which is visible as fluorescence. In light emitting diode applications, self-absorption of the light is a difficult problem since it reduces efficiency. However, to produce more charge carriers for water splitting and therefore more hydrogen, this issue was used as a benefit. In the QuAL technique, Mn-doped CdS quantum dots are put together in the titania mesopores using the SILAR method. Pt or Ni–Cu alloy was employed as a cocatalyst. In this instance, 16 mL (10.5 mL) H_2_ was seen under one sun circumstances. It should be emphasized that the device lacks expensive gold and PbS, making HER more advanced than their prior work with the Au–TiO_2_/PbS/CdS-based device [93]. The photoluminescence study provided conclusive confirmation, and Figure 8 compares its results using UV-Vis absorption spectra. A tiny and large grey color triangle displayed in Figure 8 for TiO_2_/CdS and TiO_2_/Mn-CdS, respectively, demonstrates the same for the considerable overlap in absorption and emission characteristics (recorded with 420 nm excitation source). When the excitation source used was 400 nm, white light-like emission was seen for TiO_2_/Mn–CdS. (Figure 8 inset). Figure 8 presents the possibility for self-absorption of emitted light as a secondary light source to generate additional charge carriers and therefore greater H_2_ evolution. Considering that the solid lattice is at the source of the emission, in situ light absorption is known to be beneficial and can be compared to the field effect caused by surface plasmons.

## 5. Commercial Feasibility of Solar Hydrogen

It is anticipated that the cost of producing hydrogen utilizing PEC technology will be higher (USD 10.4 per kg) than the cost of producing hydrogen from particle suspension (USD 1.6 per kg) for water splitting [94]. The high capital cost (USD 154.95 per m^2^) is blamed for the high cost hydrogen generation from PEC, whereas it is only 1.5% of PEC (USD 2.21 per m^2^) for particle suspension for a plant size of 1 ton per day (TPD) hydrogen net output. The aforementioned figures may be reviewed due to the vast and inconsistent light absorption with films/panels and powder/suspension, respectively. However, it should be noted that just 19.8% of the total capital cost is contributed by particles, compared to 89.6% for the production of PEC cells. It is well known that PEC technology is more costly than particle suspension since the former consists of many layers of various materials such as semiconductor layer(s) for light absorption, co-catalyst for charge usage to produce products, interface layers to provide contact between semiconductor and co-catalyst, and then properly integrating them for effective water oxidation. Additionally, the preparation procedure for the photoelectrode requires the use of sophisticated/expensive methods such as atomic layer deposition (ALD) and molecular beam epitaxy (MBE) in order to produce the layers of photoelectrode materials with correct contact among them. Although particle suspension can produce hydrogen at a low cost, there are still several obstacles to overcome: (i) lack of knowledge about how solar flux is used by the particles due to light scattering and poor light absorption coefficient; (ii) plant functioning is questionable due to safety concerns brought up by the cogeneration of H_2_/O_2_ gases, and (iii) Poor solar light penetration into the deeper particles in the bed causes the system to work poorly. It is advised to use particle catalysts in the form of films for efficient light use in order to solve several of the issues mentioned above. Furthermore, the creation of thin films of active particles does not require a sophisticated infrastructure, therefore producing hydrogen would be much less expensive attributable to minimal capital costs.

Even if the PEC technique for solar hydrogen generation with 10% STH may be competitive with traditional methane reforming, PEC-WS must first clear a number of scientific and technological hurdles before it can be considered for large-scale operation [95]. Below are a few of them:

(i) It is necessary to increase the light absorption by using materials with narrow band gaps and by covering the photoelectrode with one more light-absorbing layer; (ii) antireflective coating must be used to decrease light reflection from the photoelectrode; (iii) in order to decrease recombination, it is necessary to tailor the nanostructural characteristics and doping levels; (iv) surface reconstruction is required, and passivation layers must be designed to reduce surface recombination; (v) the selection of the proper materials with the required energetics for effective charge separation; (vi) in order to solve the stability problems, designing protective layers, adjusting electrolyte compositions, and tuning semiconductor architectures are all important; (vii) Due to a sizable number of extremely regulated experimental settings and strict vacuum requirements, the procedures for producing photoelectrodes on a commercial scale are exceedingly difficult. It is important to note that, with the exception of the antireflective coating (ii) and the fabrication of the electrodes (vii), most of these barriers (i, iii, iv, and v) are also relevant to thin film photocatalysis. Additionally, the economically advantageous SILAR approach provides the opportunity to construct light-absorbing components in the pores of wide band gap semiconductor, decreases charge recombination, and only partially answers point (iv).

According to Jacobsson et al., the PEC of water splitting may soon become obsolete, since significant advancements in silicon PV technology have rendered the development of PEC-WS systems no longer worthwhile [91]. The cost of producing hydrogen is significantly higher even with a 10% STH conversion efficiency compared to solar PV technology with a 15% conversion, which is a key argument against the PEC-WS. Finding a photo-absorber with all the required characteristics, such as a proper bandgap, band edge locations, and stability, which drive all the functions, from light absorption to catalytic reaction, is the optimum situation for a functional PEC-WS technology. Finding a photo-absorber with stable hydrogen generation is exceedingly challenging in practice. It should be noted that, due to lower material and equipment costs compared to the latter, thin film photocatalysis, which has an efficiency of 3–4%, may be economically comparable with PEC-WS, which has an efficiency of 10% for STH conversion. Although PEC uses a thin sheet approach, the utilization of bias and complex manufacturing techniques raises the price of such devices. It must be stressed that a simplistic strategy such as artificial leaf is likely to produce superior outcomes with no bias imposed and uncomplicated (thin film) production techniques. Despite the fact that Jacobssen’s point of view is fascinating, Si-PV manufacture is a well-known, extremely polluting sector. Recycling of Si-PV panels will soon become a problem for the environment. In essence, many people recognize that the Si-PV technology’s “green energy” label is false. As a result, we must continue searching for true green solar technologies. For operational reasons, it is also advantageous to have rival energy conversion technologies, even those with varying price tags.

## 6. Conclusions and Prospects

The current review focused on the importance and producing economy of hydrogen as fuel. The progress based on particulate photocatalytic systems and their drawbacks also discussed. In order to move towards the economic scenario, we discussed about the advantages of thin film-based techniques and covered a wide range of options with improved solar hydrogen generation. We explored a number of strategies for enhancing photocatalytic water splitting activity. Apart from activity, thin-film (or panel) versions of particles have previously been shown to be scalable and would undoubtedly be a better form of the catalyst. In comparison to their powder counterparts, the films have the ability to produce hydrogen at levels up to two orders of magnitude greater. The following are the causes: (a) efficient light absorption with minimal light scattering; (b) the use of charge carriers on a small scale in a few hundred square nanometers for water splitting to produce hydrogen. The charge carriers produced in solar cells and DSSCs must travel across a distance of several microns (8–14 μm) in order to produce current. In this regard, it is important to note that the large-sized photocatalyst panels discussed in the current research may offer a different and sustainable method of producing hydrogen. The fabrication of huge photocatalyst panels is inexpensive and has the unique benefit of local charge carrier usage, unlike photoelectrodes which need highly expensive technology. It should be observed that the H_2_ generation between OWS and with sacrificial agent differs by 3–4 orders of magnitude, with the latter showing the high rate of H_2_ production. Glycerol is a plentiful chemical that may be employed for short-term purposes since transesterification produces biodiesel. Nevertheless, OWS and improving its STH effectiveness should be the main priority. It is wise to take advantage of this characteristic to speed up the pace of H_2_ generation because experiments carried out in direct sunlight raise the temperature of the catalyst and solution. In any scenario, research on water splitting should ultimately be performed in full sunshine. Utilizing the 4–5% UV light that is found in sunshine is also crucial. Overall price reduction is ultimately being driven by increased device efficiency. Therefore, we advise photocatalysis experts to conduct their upcoming studies using thin films under direct sunlight. Although nanoscience and materials science have advanced significantly over the last several decades, it is still a challenge to employ the findings in a way that is appropriate for solar hydrogen production by photocatalysis. In this overview, certain achievements are emphasized, including the in situ assembly of light-absorbing semiconductor quantum dots in substrate pores, which results in effective charge separation via heterojunctions. We believe that by using nanoscience and nanotechnology wisely, the enormous gap in time scales between photophysical and photochemical processes might potentially be resolved. Therefore, coordinated efforts are necessary, and it is probable that they will result in a solution for economically effective and scalable water splitting to produce significant amounts of hydrogen.

## Figures and Tables

**Table 1:** A few highly efficient reported photocatalytic systems for better hydrogen efficacy.

Photocatalyst	Cocatalyst	Efficiency	Reference
TiO_2_	Pt/RuO_2_	QE: 30 ± 10% at 310 nm	[23]
La_2_Ti_2_O_7_:Ba	NiO*_x_*	QE: 35% at (<360 nm)	[24]
Sr_2_Nb_2_O_7_	Ni	QE: 23% at (<300 nm)	[25]
NaTaO_3_:La	NiO	AQE: 56% at 270 nm	[26]
Ga_2_O_3_:Zn	Rh_2−_*_y_*Cr*_y_*O_3_	AQY: 71% at 254 nm	[27]
GaN:Mg/InGaN:Mg	Rh/Cr_2_O_3_	AQE: 12.3% at 400–475 nm	[28]
CDots-C_3_N_4_		AQE: 16% at 420 nm	[29]

**Table 2:** The highest recorded solar hydrogen production using several photocatalyst systems that have been thoroughly studied in powder form.

Photocatalyst	Co-Catalyst	Sacrificial Agent	Light Source	H_2_ Yield (mmol h^−1^·g^−1^)	Catalyst wt. (mg)	Stability (h)	Ref
ZnS–In_2_S_3_–CuS_2_	—	0.1 M Na_2_S + 1.2 M Na_2_SO_3_	300 W xenon lamp (UV-cut off filter, λ > 420 nm)	360	10	9	[61]
TiO_2_/CdS	Pt	Lactic acid	300 W xenon lamp (λ > 400 nm)	128.3	50	15	[62]
CdS nanowires	MoS_2_	Lactic acid	300 W xenon lamp (UV-cut off filter, λ > 420 nm)	95.7	20	24	[63]
CdS@TiO_2_	Pt	0.1 M Na_2_S + 0.02 M Na_2_SO_3_	Sun light	44.8	10	24	[64]
CdS	MoS_2_	Ethanol	300 W Xe arc lamp (λ > 420 nm)	140	10	150	[65]
CdS	CoP	Lactic acid	300 W Xe arc lamp (λ > 420 nm)	106	20	18	[66]
CdS	MoS_2_	Lactic acid	300 W Xe arc lamp (λ > 420 nm)	49.8	200	24	[67]
CdS–titanate	Ni	Ethanol	Sun light	31.82	100	15	[68]
ZnS–In_2_S_3_–Ag_2_S	—	0.6 M Na_2_SO_3_–0.1 M Na_2_S	300 W Xe arc lamp (λ > 420 nm)	220	15	16	[69]
TiO_2_	Au–Pt	Methanol	One sun condition	6	20	125	[70]

**Table 3:** Highest recorded solar hydrogen production using several photocatalysts that were tested in powder form at low concentrations.

Photocatalyst	Co-Catalyst	Sacrificial Agent	Light Source	Hydrogen Yield (mmol h^−1^·g^−1^)	Mass (mg)	Stability (h)	Ref.
CdS nanorods	Ni_2_P	Na_2_S–Na_2_SO_3_	300 W Xe lamp (λ > 420 nm)	1200	1	12	[72]
CdS nanorods	Co_x_P	Na_2_S–Na_2_SO_3_	300 W Xe lamp (λ > 420 nm)	500	3	25	[73]
CdS/ZnS	—	0.5 M Na_2_SO_4_	300 W Xe lamp (λ > 400 nm)	239	1	12	[74]
CdS nanorods	FeP	Lactic acid	LED: 30 × 3 W, λ > 420 nm	202	5	100	[75]
CdS nanorods	Cu_3_P	Na_2_S–Na_2_SO_3_	300 W Xe lamp (λ > 420 nm)	200	1	12	[76]
CdS nanorods	WS_2_	Lactic acid	300 W Xe lamp (λ > 420 nm)	185.8	1	50	[77]
CdS nanorods	Co_3_N	Na_2_S–Na_2_SO_3_	300 W Xe lamp (λ > 420 nm)	137.3	1	48	[78]
CdS nanorods	PdPt	Lactic acid	150 W Xe lamp	130.3	1	20	[79]
CdS/MoS_2_	—	Lactic acid	Sunlight	174	1	25	[80]
MoS_2_-RGO-CoP/CdS	MoS_2_/CoP	Lactic acid	Sunlight	83.9	1	20	[81]

**Table 4:** Overview of photocatalytic activity existed in thin film as well as powder form.

Photocatalyst	H_2_ Evolution-mmol h^−1^·g^−1^.		Reference
	Powder Form	Thin Film Form	
Au–Pd/rGO/TiO_2_	0.50	21.5	[56]
BiVO_4_ and SrTiO_3_:La,Rh	0.8	4.5	[41]
(RhCrO_x_/LaMg_1/3_Ta_2/__3_O_2_N/(Au,RGO)/BiVO_4_:Mo)	0.11	0.45	[84]
LTC/Au/BiVO_4_	Nil	0.47	[43]
Ga-LTCA/Au/BiVO_4_	Nil	2.2	[43]
Al-SrTiO_3_/RhCrO_x_	Nil	5.6	[36]
Pt@mp–gC_3_N_4_	0.08	0.6	[35]
Pd/TiO_2_	9.1	30	[34]
Cu–Ni (1:1)/TiO_2_	1.75	41.7	[92]
NEC/WS_2_/CF	4.9	64.85	[93]
Au−Pd/C/TiO_2_	0.48	6.42	[56]

## Data Availability

Not applicable.
